# Volunteering among pre-clinical medical students: Study of its association with academic performance using institutional data

**DOI:** 10.12688/mep.19105.1

**Published:** 2022-03-30

**Authors:** Laila Alsuwaidi, Leigh Powell, Deena Alhashmi, Amar Hassan Khamis, Nabil Zary

**Affiliations:** 1 College of Medicine, Mohammed Bin Rashid University of Medicine and Health Sciences, Dubai, United Arab Emirates; 2 Institute for Excellence in Health Professions Education, Mohammed Bin Rashid University of Medicine and Health Sciences, Dubai, United Arab Emirates; 3 Hamdan Bin Mohammed College of Dental Medicine, Mohammed Bin Rashid University of Medicine and Health Sciences, Dubai, United Arab Emirates

**Keywords:** student volunteering, service-learning, academic performance, medical education

## Abstract

**Background: **Participating in volunteering activities during students’ higher education experience is becoming more commonplace. Studies have noted that volunteering has a positive impact on the academic performance of undergraduate medical students. However, the majority of these studies rely on self-reported data like surveys, interviews, and journals. In this study, we leverage actual institutional data to examine the relationship between volunteering and academic performance among medical students in the pre-clinical phases of the Bachelor of Medicine and Bachelor of Surgery (MBBS) program. The current study also explores the factors that might influence the studied relationship.

**Methods: **A retrospective analysis was conducted in the College of Medicine at the Mohammed Bin Rashid University of Medicine and Health Sciences (MBRU) in Dubai, United Arab Emirates. Three years of volunteering records for three cohorts of undergraduate medical students enrolled in the MBBS program between 2016 – 2018 were reviewed and analyzed to complete this study. In addition, the correlation between the Annual Grade Point Average (AGPA) and volunteering was studied, and the regression coefficients for the two variables were observed across the three cohorts in each study year.

**Results: ** Analysis of 153 undergraduate medical students' volunteering records revealed a significant positive relationship between the AGPA and the number of volunteering in year two. The correlation was insignificant in year one, year three, and across the three cohorts.

**Conclusions: ** The association between academic performance and volunteering among undergraduate medical students appeared to be positive. However, this relationship differs across the pre-clinical study years in medical school and is likely influenced by factors that impact students’ motivation towards volunteering. Therefore, having the volunteerism program embedded in curriculum planning is an aspect to be explored to enhance medical students’ learning and professional development through volunteerism.

## Introduction

Participating in volunteering activities during students’ higher education experience is becoming more commonplace. Volunteering has been defined as “an intentional behavior, carried out without being a professional obligation and aimed at supporting, preserving and promoting social values, without waiting for any moral or material rewards from others” ^1^. While opportunities for students to volunteer may come from places within the community; many volunteering opportunities are offered as part of the university setting itself. In their study of 2,309 undergraduate students, Astin and Sax noted that the majority (51.8%) of students who volunteered did so within their university ^2^. Students volunteer for a variety of reasons, motives, and benefits. These can include a desire to acquire knowledge, develop new skills, participate in experiences that benefit their education and/or careers, and enhance their academic learning ^1, 2^.

Volunteering has been shown to positively impact undergraduate medical students, contributing to better psychological health, wellness, and personal development ^3– 7^. Medical students may participate in various volunteering activities such as providing direct clinical service in public health interventions, holding awareness sessions for the public, providing administrative support for local health authorities, or even volunteering for crisis response ^8, 9^. In return, students gain distance from the many stressors of medical school. Studies noted that students’ involvement in leading and organizing extracurricular activities resulted in lower burnout levels and helped develop stress management skills ^4, 10– 12^. Participating in volunteer community service projects has been noted to promote leadership and teaching skills, and helps to build student awareness of health needs in their community ^5^. Volunteering has also been shown to promote the development of competencies related to soft skills, such as improved communication, decision-making, social skills, and higher levels of empathy ^5, 10, 13^.

Within the formal academic environment, students studying to become health professionals must meet specific competencies based on skills, traits, and behaviors required to be an effective health professional. Competency-based frameworks, such as those laid out by the American Council for Graduate Medical Education (ACGME) and the CanMEDS framework from Royal College of Physicians and Surgeons of Canada, assess competencies that take a holistic look at practice, focusing not just on medical knowledge and skills but also interpersonal communication, data literacy, professionalism, leadership, and advocacy ^6, 7^. Health professions programs have the challenge of integrating learning opportunities to help build these diverse competencies into an already packed curriculum. There exists the potential for volunteering activities to supplement the learning of competencies to enable students to put their professional skills and knowledge to work for the community good while simultaneously promoting the development of competencies.

While volunteering has been shown to have positive benefits for students, it is essential to understand how volunteering may impact academic performance. Studies have noted that volunteering positively impacts academic performance, as indicated by improved overall Grade Point Average (GPA) and student success ^12– 14^. As success in educational performance significantly affects students’ self-esteem and motivation in higher education, educators and researchers need to identify and understand what factors impact academic performance ^15, 16^.

This study will help to inform the literature on volunteering and how it affects undergraduate medical students’ academic performance. While preceding studies have reported findings in this area, most rely on self-reported data like surveys, interviews, and journals. This study is unique in that it leverages actual, institutional data to take a quantitative approach in examining the relationship between volunteering and academic performance across three cohorts of medical students in pre-clinical phases of the Bachelor of Medicine and Bachelor of Surgery (MBBS) program.

This research study aims to examine the association between volunteering and the academic performance of undergraduate medical students. Accordingly, our research questions are:

- What is the relationship between Annual Grade Point Average (AGPA) and student volunteering?
- What are the factors that possibly influence the relationship between volunteering and AGPA?

## Methods

### Ethics approval and consent to participate

Ethical approval for the study was granted by the Mohammed Bin Rashid University of Medicine and Health Sciences (MBRU) Institutional Review Board (Reference #: MBRU-IRB-2017-003). Further clarification can be obtained from the MBRU-IRB at irb@mbru.ac.ae. 

All methods were performed following the relevant guidelines and regulations (Declaration of Helsinki). No students were enrolled for this study hence informed consent was waived off by the MBRU-IRB. No questionnaire or survey was separately created or designed for this study. This was indicated in the IRB application that was submitted to MBRU-IRB, which approved the waiver.

### Study context

During the five years in which the College of Medicine at the MBRU in Dubai has been in existence, there has been a steady increase in the number and variety of volunteering activities offered to students studying in its Bachelor of Medicine and Bachelor of Surgery (MBBS) program. The MBRU, six-year MBBS program is divided into a one-year basic sciences phase (year one), a two-year organ system phase (years two and three), and a three-year clinical sciences phase (years four, five, and six). Student progression to the next phase is subject to successful completion of the progression requirements and a minimum cumulative Grade Point Average (cGPA) at the end of the preceding phase. Although volunteering is not required for graduation, it is highly encouraged for students to give back to their community while building confidence and helping students develop competencies needed for their future practice as medical doctors. To examine how volunteering activities might be used to supplement the curriculum, it is first essential to understand the relationship between academic performance and volunteering and explore the factors that influence this relationship.

### Study design

A retrospective analysis was conducted in the College of Medicine at the MBRU in Dubai, United Arab Emirates. The study population includes three cohorts of medical students enrolled in the MBBS program between 2016 – 2018. Since the study was intended to investigate the relationship between the medical students’ academic performance and their level of engagement in volunteering, the study was conducted using a correlation research design.

### Data collection and analysis

Three years of the volunteering records for the three MBBS cohorts were used to complete this study. The volunteering activities were retrieved from the students’ volunteering record as recorded by the university and the Annual Grade Point Average (AGPA) for each student was retrieved from the Student Information Self-Service (SIS). AGPA and volunteerism data was retrospectively collected. The confidentiality of information gathered from the participants’ records was preserved. 

A quantitative approach was adopted as the study was based on variables measured with numbers and analyzed with statistical procedures. The measure of volunteering activities is based on the number of volunteering events each student participated in as recorded by the university, and the measure for academic performance is AGPA. Spearman's rho non-parametric test was used to measure the strength of association between the two variables, where the value r = 1 means a perfect positive correlation and the value r = -1 means a perfect negative correlation. All data from these records were transferred to Microsoft Excel. Standard data entry and quality control procedures were used, including double entry, range and consistency checks, and manual review of outliers. All statistical analyses were performed using IBM-SPSS software (version 25.0). We also used linear regression analysis to estimate the relationships between the two variables. Regression coefficients for the two variables were observed across the three cohorts in each study year.

## Results

In this study we reviewed the volunteering records of 153 medical students. The studied population includes more females (n = 117 out of 153, 76.5%) than males (n = 36 out of 153, 23.5%) and reflected that the majority of students participated in volunteering activities (n=132 out of 153, 86.3%). Participants’ characteristics are presented in Table 1.

**Table 1.** Description of the study sample by study year and cohort, n=153.

Study year	Cohort	Male	Female	UAE	Non-UAE	Non-volunteering	Volunteering	Total
**Year 1**	**Total** First cohort Second cohort Third cohort	**36 (23.5%)** 15 (27.8%) 8 (21.6%) 13 (21.0%)	**117 (76.5%)** 39 (72.2%) 29 (78.4%) 49 (79.0%)	**50 (32.7%)** 11 (20.4%) 15 (40.5%) 24 (38.7%)	**103 (67.3%)** 43 (79.6%) 22 (59.5%) 38 (61.3%)	**21 (13.7%)** 0 (0%) 11 (29.7%) 10 (16.1%)	**132 (86.3%)** 54 (100%) 26 (70.3%) 52 (83.9%)	**153 (100%)** 54 (100%) 37 (100%) 62 (100%)
**Year 2**	**Total** First cohort Second cohort	**23 (25.3%)** 15 (27.8%) 8 (21.6%)	**68 (74.7%)** 39 (72.2%) 29 (78.4%)	**26 (28.6%)** 11 (20.4%) 15 (40.5%)	**65 (71.4%)** 43 (79.6%) 22 (59.5%)	**24 (26.4%)** 10 (18.5%) 14 (37.8%)	**67 (73.6%)** 44 (81.5%) 23 (62.2%)	**91 (100%)** 54 (100%) 37 (100%)
**Year 3**	**Total** First cohort	**15 (27.8%)** 15 (27.8%)	**39 (72.2%)** 39 (72.2%)	**11 (20.4%)** 11 (20.4%)	**43 (79.6%)** 43 (79.6%)	**5 (9.3%)** 5 (9.3%)	**49 (90.7%)** 49 (90.7%)	**54 (100%)** 54 (100%)

### Relationship between AGPA and volunteering

Table 2 and Table 3 display the statistical analysis performed to measure the strength of the relationship between volunteering and academic performance using Spearman's rho non-parametric test. The measure of volunteering activities is the number of volunteering events recorded for each student, and the measure of academic performance is AGPA.

**Table 2.** Statistical analysis of the annual grade point and number of volunteering non-parametric correlations per study year.

Study year Spearman’s rho non-parametric test		Correlation Coefficient	*Sig. (2-tailed)	Number of students	Number of offered activities
Year1	AGPA/ Event	-0.024	0.769	153	87
Year2	AGPA/ Event	0.209	**0.047**	91	69
Year3	AGPA/ Event	0.194	0.159	54	47

**Table 3.** Statistical analysis of the annual grade point and the number of volunteering non-parametric correlations per cohort.

Cohort Spearman’s rho non-parametric test		Correlation Coefficient	*Sig. (2-tailed)	Number of students	Number of offered activities
Cohort 1	AGPA/ Event	-0.062	0.654	54	87
Cohort 2	AGPA/ Event	0.236	0.430	37	69
Cohort 3	AGPA/ Event	0.160	0.215	62	47

AGPA: annual grade point average; Event: number of volunteering per student. *. Correlation is significant at the 0.05 level (2-tailed).

The results in Table 2 revealed a significant positive relation between the AGPA and the number of volunteering in year 2 (r = 0.209; p-value is 0.047). In year 3 (r = 0.194; p-value is 0.159) the correlation was positive but insignificant and in year 1 (r = - 0.024; p-value is 0.769) the result shows negative insignificant association. Furthermore, the correlation coefficient between the AGPA and number of volunteering activities was tested across the three cohorts, reflecting a positive insignificant correlation in cohort 2 (r = 0.236; p-value is 0.430) and cohort 3 (r = 0.160; p-value is 0.215), while in cohort 1 there was negative insignificant correlation (r = - 0.062; p-value is 0.654) ( Table 3).

**Figure 1.**
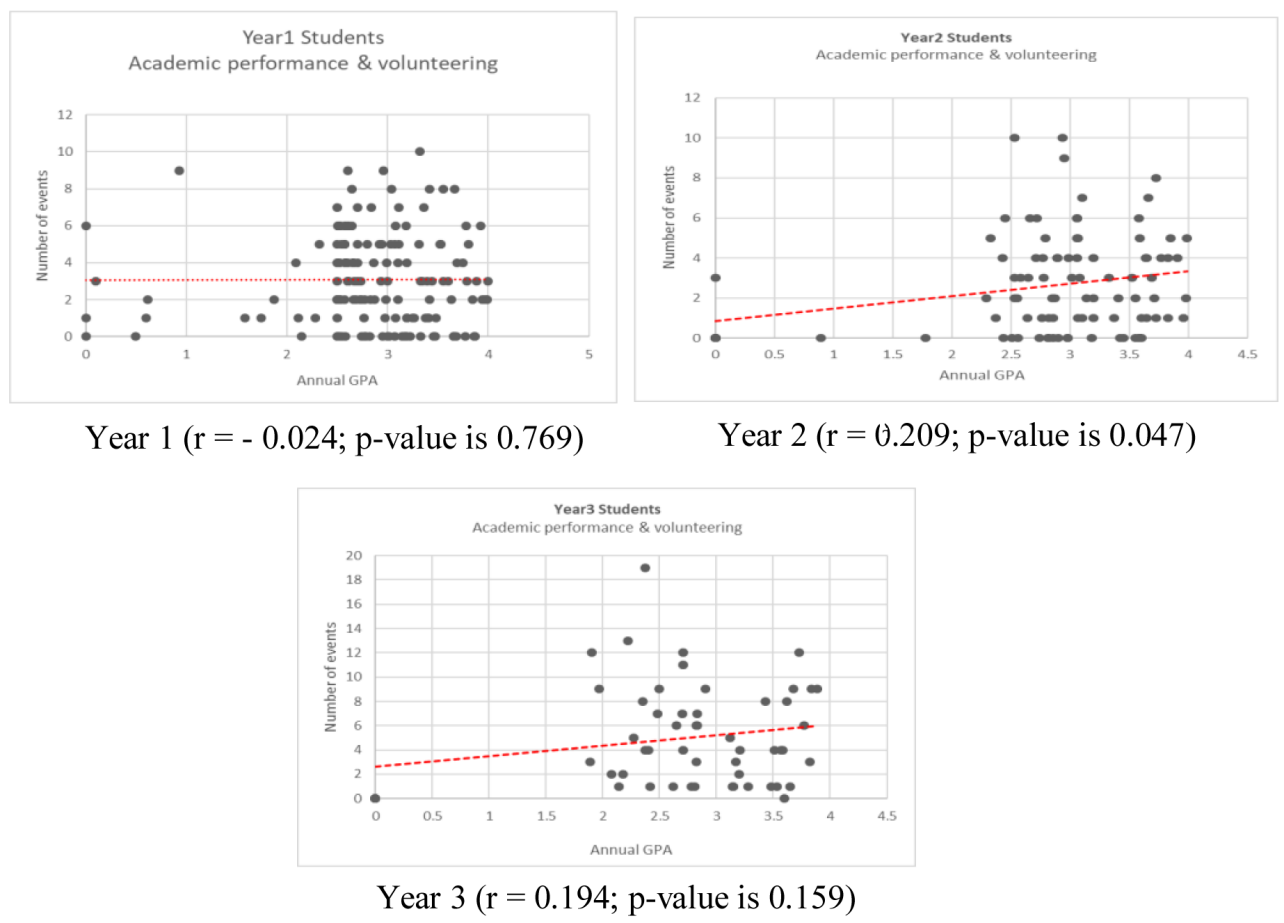
Correlation of annual grade point and number of volunteering for year 1, year 2, and year 3 students.

**Figure 2.**
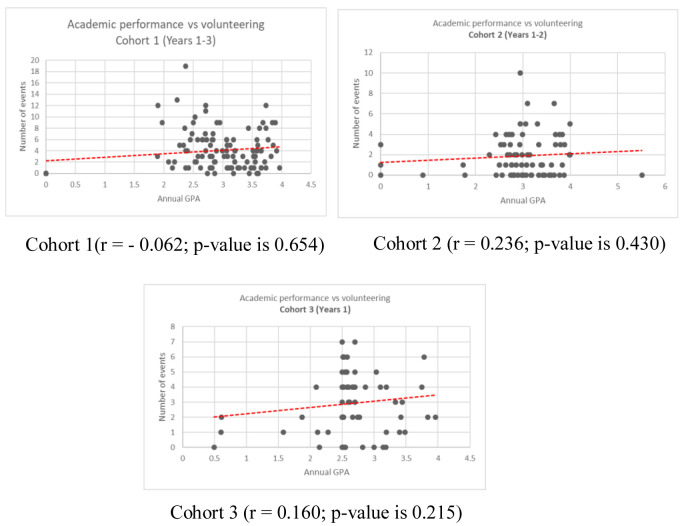
Correlation of annual grade point and number of volunteering for Cohort 1, Cohort 2, and Cohort 3 students.

The correlation between the AGPA and the number of volunteering activities across the three cohorts and within different study years is demonstrated in the scatter plots, and confidence levels for all regressions are noted in the results. ( Figure 1 and Figure 2)

### Factors that influence the relationship between volunteering and AGPA

The results in Table 4 show a statistically significant difference (p-value = 0.009) in volunteering practices between students who are UAE Nationals (4.64 (±0.79)) and students who are non-UAE Nationals 7.41(±0.68). The analysis also shows that the mean for the female and male number of volunteering was 6.41 (±0.62) and 6.81(±1.06), respectively. This was statistically insignificant (p-value = 0.756).

**Figure 3.**
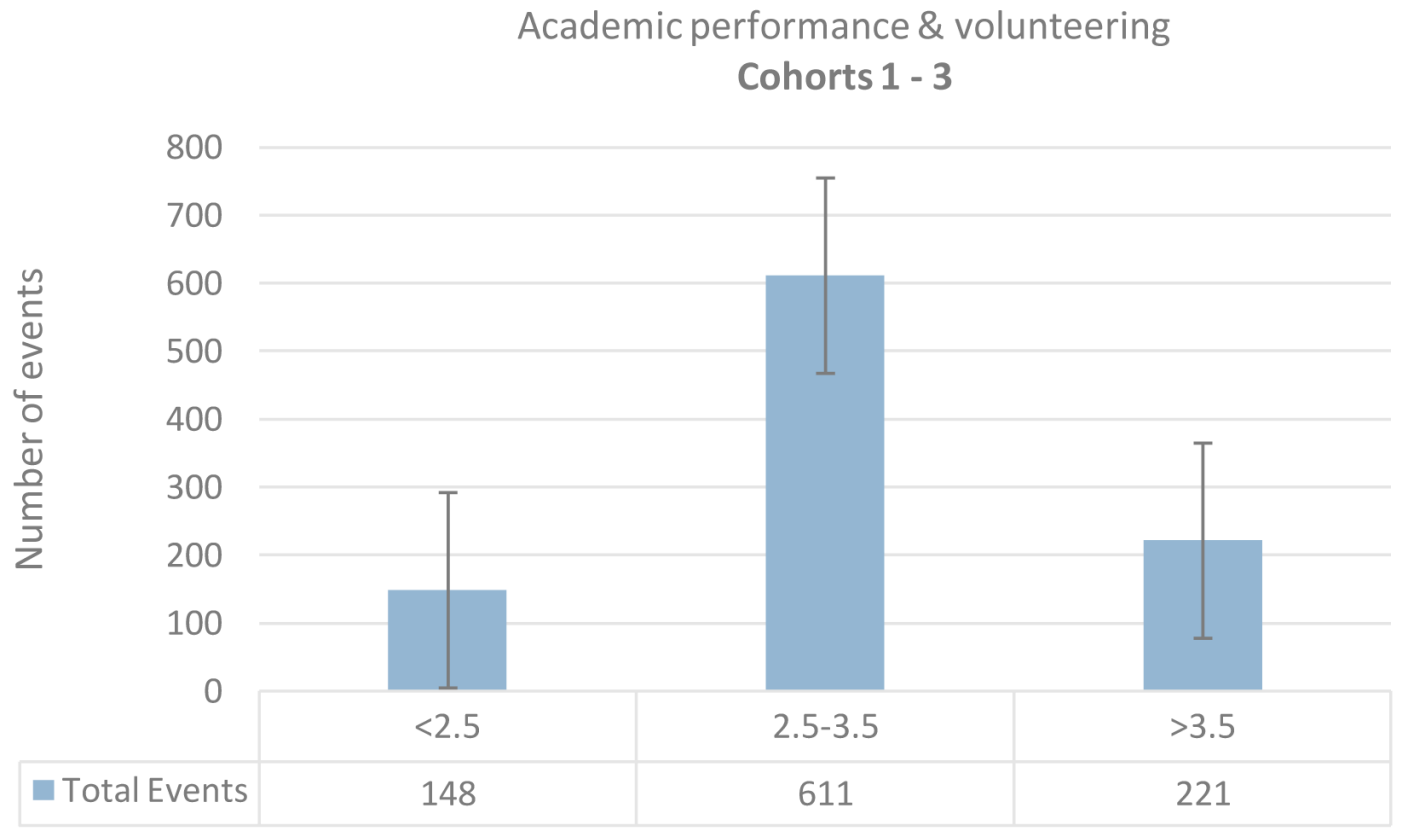
Bar chart illustrates the spread of the three cohorts’ data between three annual Average Grade Point categories.

The total number of volunteering events for students with suboptimal performance, optimal performance, and high performance is 148, 611, and 221, respectively. The distribution of the total volunteering activity and AGPA for each cohort is illustrated in Figure 3. Students with optimal performance (AGPA 2.5 - 3.5) volunteered more frequently than students with either suboptimal performance (AGPA < 2.5) or high-performance (AGPA > 3.5).

**Table 4.** Cross-sectional analytical association of the nationality/gender and number of volunteering.

a. Volunteering vs. gender						
	**Gender**	N	Mean	Std. Deviation	Std. Error Mean	*Sig.
Num. Volunteering	Female	117	6.4103	6.73277	.62245	p-value = 0.756
	Male	36	6.8056	6.37773	1.06295	
						
b. Volunteering vs. nationality						
	**Nationality**	N	Mean	Std. Deviation	Std. Error Mean	*Sig.
Num. Volunteering	UAE	50	4.6400	5.60889	.79322	p-value = 0.009
	Non-UAE	103	7.4078	6.92174	.68202	

*P-value of 0.05 was used as a level of significant

## Discussion

This study quantitatively examined the relationship between volunteering and academic performance in the pre-clinical phase of undergraduate medical education. Unlike many previous studies that rely on student self-reported data, our study was able to use actual institutional data to understand this relationship. This research also contributes to the growing pool of research being conducted in the UAE, a unique factor given that much of the research on this topic reports findings from North America and Europe.

### Relationship between volunteering and AGPA

The results of the current study suggested a positive correlation between AGPA and volunteering among medical students. However, this relationship differs across the three pre-clinical study years. In year one, the relationship was negative (r = - 0.024), while in years two and three, the relationship was positive (r = 0.209 and r = 0.194 respectively). The positive correlation was only found significant in year two (p-value is 0.047). This observation concurs with Tinto (1993), who studied students’ decisions to volunteer, and reported that the students’ engagement in volunteering might be impacted by the transition from high school to college. This is potentially based on factors such as the consistency in norms and expectations in past environments as well as how prepared students feel to navigate participation in a new environment ^17^. A students first year in a medical college is marked by a transition phase from high school to college where the uncertainty level among the students is elevated ^18^. Students may not be immediately aware of how to strike a balance between academics and non-academics in order to maintain academic standing. First year students also have the added pressure of ensuring they fulfill the progression requirement of a minimum AGPA mandated in the degree plan of the MBBS program, hence there may be a greater reluctance to volunteer.

An insignificant negative correlation was reported in the Cohort 1 (r = -0.062; p-value is 0.654). The students in Cohort 1 are unique in that they were the first cohort of students in the newly founded university, who are nicknamed “The Pioneers”. There is a high likelihood that these students had the same degree, or even greater degree, of uncertainty, which could have reduced students’ motivation to volunteer. This may aid to elucidate the obtained negative correlation between the two variables in year one and first cohort students. Cohort 1 also had access to a limited number of volunteering activities in the early years, with more competition over these limited slots, which could also explain the insignificant negative relationship between the two variables. 

A positive insignificant correlation was observed across Cohort 2 and Cohort 3 (r = 0.236; p-value is 0.430 and r =0.160; p-value is 0.215 respectively). The positive relationship in later cohorts and years two and three could support the claim that students are more assured of their environment after year one and feel more empowered to volunteer while maintaining achieved AGPA. Despite the assuring environment, the positive relationship was not significant, except in year two. Holdsworth (2010) explored how student motives to volunteer change over time, noting “opportunity” as one motivation contributing to whether or not a student would volunteer their time. Opportunity is defined as “captur(ing) circumstances that students find themselves in”, which may include things like commitments to external activities and having more spare time ^19^. It is quite possible that students in Year 2 find themselves with more opportunities to volunteer because it is the only year out of the first three that does not have high stakes and a high-pressure end-of-year bar exam.

The types of volunteering activities on offer could also be a factor. It is noteworthy that most activities available across the three study years were primarily focused on interpersonal and communication skills. Year three students may not find these activities of great interest as their motives for volunteering may shift towards activities that build hands-on skills in preparation for clinical placements. Skills building was noted as a motive for students in volunteer in Holdsworth (2010), and Handy *et al.*, (2010) describing that initial motives for students to volunteer is often as a way to contribute to the development of their resume ^19, 20^. Shifting more focus on the kinds of volunteering activities the university provides may help to see greater gains in terms of volunteering and its impact on students. Medical students can be provided with experiential learning opportunities beyond the traditional medical school curriculum, particularly intergenerational interactions and alike community-oriented experiences. These experiences enhance essential communication skills and promote an increased awareness of the contextual health problems faced by community members ^21^.

### Influencing factors

Students on the higher and lowers ends of academic performance, as expressed by AGPA, volunteered less frequently than students with optimal performance. Our results do not agree with the findings of previous literature, which suggested that students with higher levels of service/service-learning reported higher grades, attendance, and other academic success outcomes ^22, 23^. There are many factors which could contribute to this finding. Students may not have had equal opportunities to build skills or personality traits conducive to creating the harmonious work-life integration critical to improving physical, emotional, and mental well-being and ultimately improvement of their career ^24^. It is also possible that students on the highest and lowest ends of academic performance have differing priorities when it comes to volunteer, where students with lower AGPAs may be more concerned about using time to improve academic standing, and students with higher AGPAs may be naturally more focused on academic aspects.

### Characteristics of students who volunteer

The results of this study show that there is equal participation in volunteering from a gender perspective, with both female and male students exhibiting a similar level of involvement in volunteering activities (mean of volunteering number 6.41±0.62 and 6.81±1.06 respectively; p-value = 0.756), though the percentage of females (76.5%) among the studied population is far more than that of the males (23.5%). Additionally, the level of participation in volunteering activities among non-UAE nationals was significantly higher than that of the UAE nationals. Given the limited amount of research in this area using the UAE as a context, it is difficult to account for why non-UAE nationals volunteer more than UAE nationals. However, Astin and Sax found that the predisposing factor for volunteering was that the student had volunteered while in high school ^2^. The UAE has a unique cultural landscape featuring a diversity of high school curriculum, with many attending private schools and some UAE nationals attending government schools. A further area of exploration could include taking a deeper look at students’ high school context to determine better if the student had previous volunteering experience or if certain high school curriculums promote volunteering more than others. It is also possible that connections to home, such as a social and familial support structures, might play a role in a student’s willingness or availability to volunteer. Further study could examine this more closely to understand the demographics of non-UAE nationals who grew up in the region versus those who re-located for university.

### Limitations

This study has several limitations. Any volunteering activities students might have participated in outside of the university are not necessarily reflected in the students record, unless directly reported, and so are not factored into the records used for this study. Additionally, the data in this study constitutes a sample from a single medical school in the UAE. It would be worthwhile to conduct follow-up studies that compare several programs across multiple institutions, both in the UAE and out, as well as to extend the research to include the clinical phases of the medical program. Finally, the volunteering opportunities themselves should be look at more closely to understand how they support the student and the curriculum. A case study of students in the UK suggests that both students and stakeholders recognize that the promotion of volunteering should seek to align institutional practices to promote and support volunteering with young people's expectations and aspirations ^25^. Volunteering is a long-term commitment that should be approached through a motivational orientation. It has been described that work values and community culture as well as the dispositional factors of a student, such as personality traits, beliefs, and values are those which influence a student’s decision to volunteer ^26, 27^. 

### Future work

Further research directed to test students’ personality traits related to volunteering motivation and motivational orientation (extrinsic/ intrinsic) are required to provide an indication of the functional motives that are most salient to students and inform in the design of a rewards system that could motivate students to volunteer further. Moreover, medical schools can seek to explore how volunteering opportunities can promote the development of competencies and values, such as encouraging intergenerational volunteerism by emphasizing a culture of community involvement; connecting students with volunteer opportunities that are in alignment with the phase of their educational development; and providing the guidance necessary to create new volunteer initiatives, including financial and promotional support. Exploring the development of an institutional infrastructure to promote student volunteerism would benefit the community population and help to empower and provide learning experiences for a vulnerable student population during times of extraordinary uncertainty, such as with the COVID-19 pandemic. Further studies can inductively explore the extent of alignment between volunteering and degree plans to understand antecedents to academic performance (e.g., volunteering variables) that could play a moderating or mediating role in the correlation studied in this research.

## Conclusion

This study reported a positive association between academic performance and volunteering among undergraduate medical students. However, this relationship differs across the pre-clinical study years in medical school and is likely influenced by factors that impact students’ motivation towards volunteering. Based on these findings, students’ motivating factors towards volunteerism should receive more attention from educational institutions. Additionally, the institutional infrastructure to promote student volunteerism should be embedded in curriculum planning and assessment. More emphasis should be placed on the quality of involvement in extracurricular activities rather than the quantity. Research directed to test students’ personality traits related to volunteering motivation is required to indicate the functional motives that are most salient to the students and guide in designing a rewards system that can motivate them to volunteer further.

## Data availability

The datasets generated and/or analyzed during the current study are not publicly available as they form a part of the student academic performance record at MBRU. For the purpose of participants’ privacy protection and data confidentiality, it is stated in the MBRU-IRB that all information obtained from the participants during this study will be maintained confidentially and only accessible to the principal investigator and co-investigators of the study. However, to allow verification of published findings and to enable other researchers to build on published results, the data will be available for reviewers and other researchers by request to be sent via e-mail to the corresponding author ( Laila.Alsuwaidi@mbru.ac.ae). 
