# Supplementary material for: Volunteering among pre-clinical medical students: Study of its association with academic performance using institutional data
Source: MedEdPublish (2016). 2022 Jun 16;12:24. Originally published 2022 Mar 30. [Version 2] doi: 10.12688/mep.19105.2 (PMC9370086; doi:10.12688/mep.19105.2)
Supplement: Peer review history

# Peer review: Reviewer response for version 2

**DOI:** 10.21956/mep.20539.r32151

**Published:** 2022-07-08

**Authors:** Yan Chen

**Affiliations:** 1 Centre for Medical and Health Sciences Education, School of Medicine, University of Auckland, Auckland, New Zealand

I believe the authors have addressed my concerns and the article can be indexed as it is now.

Have any limitations of the research been acknowledged?

Partly

Is the work clearly and accurately presented and does it cite the current literature?

Partly

If applicable, is the statistical analysis and its interpretation appropriate?

Partly

Are all the source data underlying the results available to ensure full reproducibility?

Yes

Is the study design appropriate and is the work technically sound?

Yes

Are the conclusions drawn adequately supported by the results?

Partly

Are sufficient details of methods and analysis provided to allow replication by others?

Partly

Reviewer Expertise:

Quantitative research methods, assessment and evaluation, wellbeing

I confirm that I have read this submission and believe that I have an appropriate level of expertise to confirm that it is of an acceptable scientific standard.

# Peer review: Reviewer response for version 2

**DOI:** 10.21956/mep.20539.r32149

**Published:** 2022-06-29

**Authors:** Brett Vaughan

**Affiliations:** 1 Department of Medical Education, The University of Melbourne, Melbourne, VIC, Australia; 2 Faculty of Health, Southern Cross University, Lismore, NSW, Australia

Thanks to the authors for the opportunity to review the revised manuscript. The changes made to the manuscript have improved the work. Further, the changes are a more tempered reflection of the results.

Have any limitations of the research been acknowledged?

Yes

Is the work clearly and accurately presented and does it cite the current literature?

Yes

If applicable, is the statistical analysis and its interpretation appropriate?

Partly

Are all the source data underlying the results available to ensure full reproducibility?

Yes

Is the study design appropriate and is the work technically sound?

Partly

Are the conclusions drawn adequately supported by the results?

No

Are sufficient details of methods and analysis provided to allow replication by others?

No

Reviewer Expertise:

Health professions assessment & evaluation

I confirm that I have read this submission and believe that I have an appropriate level of expertise to confirm that it is of an acceptable scientific standard.

# Peer review: Reviewer response for version 1

**DOI:** 10.21956/mep.20463.r31826

**Published:** 2022-05-12

**Authors:** Brett Vaughan

**Affiliations:** 1 Department of Medical Education, The University of Melbourne, Melbourne, VIC, Australia; 2 Faculty of Health, Southern Cross University, Lismore, NSW, Australia

Thank you for the opportunity to review this work. Volunteering is an under-researched area in health professions education so work in this area is welcome.

 The Introduction largely covers the relevant literature. In the third paragraph, it would be helpful to clarify whether this is different from other health professions programs or specific to medicine. The sentence also needs to be referenced to support the assertion. At the end of this paragraph, is it specific competencies being developed by volunteering or more broadly? There should also be an element that addresses whether the volume or quality (or both) of volunteering activities is associated with improved performance.

 In the data collection, please clarify that the data were not retrospectively collected, rather the analysis was based on retrospective data.  As per the comment about the Introduction above, the study appears to be undertaken on the basis of the volume of activity. Spearman's rho provides an indication of the association between the two variables of interest but there is no indication as to how the coefficient was interpreted by the authors. This would be helpful to develop an understanding of the interpretation of the data in the Discussion.

 There are a number of analyses that are not described in the Methods (gender, UAE/non-UAE) and these should be included along with effect size calculations where appropriate. Linear regression is mentioned in the last part of the paragraph, however, there are no analyses presented that indicate it was undertaken.

 In the Results, in Tables 2 and 3, the header refers to the 'number of activities offered'. Please clarify if this means the number of activities the students participated in. Whilst the correlations were statistically significant, the rho values were trivial to low. For example, AGPA and volunteering number per year was r=0.21 - this suggests that approximately 4% of the AGPA was accounted for by the number of activities participated in.

 As mentioned in the Methods section, effect size calculations should be presented for p values that are significant. The classification of AGPA was interesting and will need to be described in the Methods along with references to support the categorisation - why was the optimal performance in the mid-range of AGPA scores?

 In the Discussion, there is a little bit of repetition with respect to the presentation of results. Further, there are only a few references to the literature throughout and this is an aspect that could be strengthened. Probably one of the more interesting elements of the study is the gender aspect of volunteering - this should be more prominent in the manuscript, although I appreciate this is not the research question.

 The Conclusions likely need to be redrawn. There was a positive association between performance and volunteering but the association was weak. Whilst I agree that "students’ motivating factors towards volunteerism should receive more attention from educational institutions" the conclusion per se is not from the study. It may be better to add that we need to understand other factors associated with volunteering that might influence GPA. Further, the authors suggest that "More emphasis should be placed on the quality of involvement in extracurricular activities." Again, I agree with the statement, however, this is the opposite of what was presented in the work and could be clarified by stating where the 'emphasis' should be rather than the quantity.

 For the references, the Holdsworth reference on page 7 needs to be amended to be consistent with the guidelines.

Have any limitations of the research been acknowledged?

Yes

Is the work clearly and accurately presented and does it cite the current literature?

Yes

If applicable, is the statistical analysis and its interpretation appropriate?

Partly

Are all the source data underlying the results available to ensure full reproducibility?

Yes

Is the study design appropriate and is the work technically sound?

Partly

Are the conclusions drawn adequately supported by the results?

No

Are sufficient details of methods and analysis provided to allow replication by others?

No

Reviewer Expertise:

Health professions assessment & evaluation

I confirm that I have read this submission and believe that I have an appropriate level of expertise to confirm that it is of an acceptable scientific standard, however I have significant reservations, as outlined above.

# Response

**Published:** 2022-05-30

**Authors:** Laila Alsuwaidi

**Affiliations:** Mohammed Bin Rashid University of Medicine and Health Sciences, United Arab Emirates

We would like to extend sincere gratitude for your time and efforts in reviewing the manuscript. We are grateful for your constructive feedback and are delighted with your interest in this manuscript. We have addressed the comments/suggestions and believe that it has strengthened our manuscript. To facilitate tracing the edits, we are attaching the latest version of the manuscript with track changes, along with providing below response to the raised comments. We genuinely hope the revised version will meet your expectations.

**Introduction: **We have reviewed the introduction section and added the relevant supporting references. According to the literatures, the competencies being developed by volunteering are those with a holistic look at practice, focusing on interpersonal communication, data literacy, professionalism, leadership, and advocacy, this is in addition to the medical knowledge and skills. We believe that quality is just as important as quantity in volunteering activities, any or both could be associated with improved performance, but we have no reference to clearly address the subject. Our current study focused on volunteering activities’ volume to explore its possible association with improved performance.

**Methods:** We have reviewed the data collection and analysis sub-section and added a statement to clarify that the analysis in our study was based on retrospective data. We also described the analysis of the volunteering among different gender and UAE/non-UAE nationals. Mann-Whitney test was used to test the means and Exact Fischer chi-test for testing dependency between categorial variables. The effect size calculation is not applicable as the selected test for analysis (Exact Fischer chi-test) remains valid for small sample sizes. Thank you for bringing the subject of the regression coefficients to our attention. In fact, we have revised the results sections and noticed that there is a discrepancy in the statement; accordingly, the manuscript has been amended to describe the scatter plotting not regression.

**Results:** The header in Tables 2 and 3 refers to the activities made available for the students and they participated in. The noted observation related to the statistically significant correlation, and trivial to low rho, highlights the impact of other possible factors (other than the number of activities that students participated in) that might be associated with volunteering and influence GPA. We found this comment very useful to improve the discussion section. Regarding the sample size calculation, it was not conducted as we used the whole number of students in the batches. To attend to your inquiry, we elaborated on the classification of the annual GPA and why the optimal performance in the mid-range of annual GPA in the methods section.

**Discussion:** We have reviewed the manuscript to address the comments related to the repetition of the results presentation in the discussion section and we expand the referencing as recommended. The gender aspect of volunteering has been elaborated on further and we added some interpretation to the discussion section considering the reviewer’s comments related to the statistically significant correlation between annual GPA and the number of volunteering activities.

**Conclusion & References:** The conclusion statement has been revised to reflect the study’s outcome in more judicious approach. References were also amended as required.

# Peer review: Reviewer response for version 1

**DOI:** 10.21956/mep.20463.r31837

**Published:** 2022-04-12

**Authors:** Yan Chen

**Affiliations:** 1 Centre for Medical and Health Sciences Education, School of Medicine, University of Auckland, Auckland, New Zealand

This paper examines the relationship between medical students' volunteering activities and their academic performance. The authors provided a synthesis of relevant literature on the positive impact that volunteering could have on medical students. The study utilised official volunteering records held by the university, thus minimising recording bias. Details were given on the research design and analysis.

 In the introduction, I would have liked to see more literature to explain why volunteering would impact academic performance. It would have been beneficial to justify using GPA as an indicator of academic performance. It is also essential for the authors to explain how GPA was calculated (e.g., was it based on their end-of-year examinations, what subjects the GPA covers, etc.). The second research question, ‘What are the factors that possibly influence the relationship between volunteering and AGPA?’, implies a moderation analysis. However, this was not the case in the current paper. This research question might be better phrased as ‘What factors influence students’ volunteering behaviours?’.

 More information is required in the methods and the results sections to allow future replication of the study and better interpretation of the results:

- For instance, why does the university hold students’ volunteering record if it is not already embedded in the curriculum? What information is registered, and whether students have received any incentives to provide such information to the university? It would have been very interesting to see the types of volunteering work students engaged in if such information was already being recorded.
- The authors conducted separate analyses for each year level and each cohort. It would have been beneficial to rule out potential differences in student characteristics between each cohort before collapsing them to conduct the year-level analysis. When it comes to cohort-based analyses, it is unclear how the authors treated data that had multiple time points (i.e., how did they collapse cohort 1’s data across three years?). I think the authors might have missed the opportunity to analyse the longitudinal trend of students’ engagement in volunteering, especially for the first cohort which had three years’ worth of data.
- How many students were included in each GPA category, as shown in Figure 3? The average number of volunteering activities could be a better measure than the total number of volunteering activities. The authors proposed regression analysis in the methods, however, they did not report the regression coefficients.
- What analysis was performed for Table 4?

The authors speculated on the non-significant relationships between volunteering and academic performance, and the current study does not provide robust evidence to support a link between volunteering and medical students’ academic performance. Therefore, more caution is needed to interpret the null findings.

Have any limitations of the research been acknowledged?

Partly

Is the work clearly and accurately presented and does it cite the current literature?

Partly

If applicable, is the statistical analysis and its interpretation appropriate?

Partly

Are all the source data underlying the results available to ensure full reproducibility?

Yes

Is the study design appropriate and is the work technically sound?

Yes

Are the conclusions drawn adequately supported by the results?

Partly

Are sufficient details of methods and analysis provided to allow replication by others?

Partly

Reviewer Expertise:

Quantitative research methods, assessment and evaluation, wellbeing

I confirm that I have read this submission and believe that I have an appropriate level of expertise to confirm that it is of an acceptable scientific standard, however I have significant reservations, as outlined above.

# Response

**Published:** 2022-05-30

**Authors:** Laila Alsuwaidi

**Affiliations:** Mohammed Bin Rashid University of Medicine and Health Sciences, United Arab Emirates

Thank you very much for the thorough review and the important comments and suggestions. We trust that by meticulously considering your valuable feedback and incorporating the necessary changes in the manuscript, we were able to raise the coherence of the synthesis and better leverage the value of our contribution. To facilitate tracing the edits, we are attaching the latest version of the manuscript with track changes, along with providing the below response to the received comments. We do hope the revised version will meet your expectations.

 We have reviewed the manuscript and added to the introduction some literature to explain why volunteering would impact academic performance. The second question of the current study has now been modified in the manuscript and reads “‘What factors influence students’ volunteering behaviours?”.

 We also added to the methods section the justification for using GPA as an indicator of academic performance and we clarified the principle for GPA calculation. More information was added to the methods and results sections to address the points raised by the reviewer.

 Student characteristics across cohorts are presented in Table 3 and Figure 2. Data that had multiple time points were treated individually, each entry point recorded and merged with the relevant year group. Example X student has 3 entries across 3 years, each entry will be added to the relevant academic year.

 As advised, we inserted the number of students in each GPA category in the results section **.**

 Figure 3 was amended to present the average number of volunteering activities instead of the total number of volunteering activities.

 Thank you for bringing the subject of the regression coefficients to our attention. In fact, we have revised the methods and the results sections and noticed that there is a discrepancy in the statement; accordingly, the manuscript has been amended to describe the scatter plotting not regression.

 In Table 4, the data normality was tested using Shapiro-Wilk test followed by Mann-Whitney non-parametric test to compare the two administered groups. We reviewed the table and added the analysis name.

 In response to the received comment, the conclusion statement has been revised to reflect the study’s outcome in a more cautious approach.

 Thank you again for the valuable comments.
